# Setting Australian research priorities for child mental health clinical trials: A Delphi study

**DOI:** 10.1177/00048674251345318

**Published:** 2025-06-24

**Authors:** Ellie Tsiamis, Anthony Jorm, Lakshmi Neelakantan, Amy Morgan

**Affiliations:** Centre for Mental Health and Community Wellbeing, Melbourne School of Population and Global Health, The University of Melbourne, Melbourne, VIC, Australia

**Keywords:** Child, research priorities, clinical trial, Delphi, mental health

## Abstract

**Objective:**

To identify Australian research priorities for clinical trials in child mental health (0−12 years).

**Methods:**

A Delphi consensus study across three rounds was carried out with child mental health professional and lived experience experts. Potential research priorities for rating by the experts were identified from a rapid review; content analysis of government strategies and targeted calls for research; and panellists’ suggestions. The highest rated priorities were then reduced to a smaller number of top priorities using a resource allocation exercise.

**Results:**

A total of 391 potential priorities were rated by 66 panellists. Panellists endorsed 75 priorities for trials covering a range of mental health interventions (promotion, prevention, assessment, and treatment), as well as overarching priorities applicable to all types of trials. The final resource allocation exercise refined these further to 12 top research priorities, including two promotion trial priorities, seven prevention and assessment trial priorities, and four treatment trial priorities.

**Conclusion:**

Results will aid the Growing Minds Australia Clinical Trials Network’s review, prioritisation, and endorsement of future clinical trials in child mental health. The findings can also be used to inform key stakeholders in child mental health on where clinical trial research efforts have focused in the past and what should be considered a priority in the future.

## Introduction

Child mental health problems in Australia are common and can have a significant impact on the wellbeing of children, caregivers, and families (Schlack et al., 2021; Scott et al., 2016). The Young Minds Matter Survey found almost 14% of Australian children aged 4−11 experienced a mental disorder in 2013−2014, the most common being Attention Deficit and Hyperactivity Disorder (ADHD) and anxiety disorders (Lawrence et al., 2015). Intervening early in childhood, or when a problem is beginning to emerge, can prevent lifelong impacts from mental ill-health (Copeland et al., 2009). Prevention and early intervention can also significantly reduce the impact of adverse childhood experiences (ACEs) on child mental health (Jones et al., 2020). In addition to treatment interventions, it is important to consider the entire spectrum of mental health interventions, including promotion and prevention, if we are to address the high prevalence and burden of child mental health problems in Australia.

Growing Minds Australia (GMA) was established in response to the need for better clinical trials on child and youth mental health interventions. It is the first Clinical Trials Network (CTN) in Australia aiming to support evidence-based interventions promoting mental health and wellbeing for children and young people (Hawes et al., 2023). While there are over 35 CTNs in Australia and New Zealand, few mental health CTNs have existed, until recently (Hawes et al., 2023). MAGNET is the National CTN for adult mental health intervention in Australia (Rossell et al., 2023). Funded by the Medical Research Future Fund Million Minds Mission (2021−2026), GMA’s goal is to transform Australia’s children and youth mental health support with real-world evidence. GMA brings together multidisciplinary practitioners, researchers, consumers, and policy makers with an interest in investigator-initiated clinical trials focusing on child and youth mental health conditions. Establishing a CTN can reduce competition and support large-scale collaboration, reflect the needs and views of stakeholders, improve the quality and scale of clinical trials, and maximise evidence translation and implementation.

Australian CTNs commonly conduct research prioritisation exercises as an important activity to determine focus areas for future trials (Australian Clinical Trials Alliance, 2019). These aim to identify research topics of importance to stakeholders through consensus-based methods. Previous research prioritisation in the mental health sector has focused extensively on adults (Banfield et al., 2018; Christensen et al., 2013; Jorm et al., 2002). Some research has been conducted on youth aged 12−25 years (Mei et al., 2020) and children (Freeman et al., 2022). However, no study has been undertaken to identify the research priorities for *clinical trials* specifically in the field of child mental health interventions. The field of child mental health spans a range of developmental phases, and services are delivered across a diverse system, including school counsellors, GPs, alternative health practitioners, child and youth mental health services, private psychologists, psychiatrists, paediatricians, as well as through online platforms. Given this diversity, it is important to prioritise where future clinical trials should focus to improve intervention research and ultimately child and family outcomes.

A review of Australian clinical trials on child mental health over the last 10 years (2013−2022) provided a picture of recent research effort and informed the research prioritisation exercise (Tsiamis, 2024). Most studies included in our review were randomised controlled trials (RCT) (77.7%) and 62.0% of which were single-site RCTs. Interventions were most commonly psychological or psychosocial and delivered by health professionals in a multi-session format. Few trials focused on select population groups, such as rural/remote communities or Indigenous peoples. When compared with the prevalence and burden of child mental health problems, the review sample identified an under-representation of clinical trials on several mental health problems, including depression, eating disorders, trauma-and stressor-related disorders, intellectual disability, and self-harm. These findings suggest some areas where future clinical trials could focus to address gaps, but it is unclear whether addressing research gaps should be the priority or whether there are other considerations that are more important, such as targeting high risk groups, implementation in real-world settings, and societal cost from child mental health problems.

This study aimed to identify research priorities for child mental health clinical trials that reflect the views of stakeholders (Professional and Lived Experience) in Australia through a phased approach. Phase 1: the Delphi study aimed to reach a consensus on what topics are research priorities across the spectrum of mental health interventions (promotion, prevention, assessment and treatment). Phase 2: the resource allocation study (value-weighting exercise) sought to distinguish the top research priorities for child mental health clinical trials, highlighting where GMA-CTN should focus on in the future.

## Methods

### Study design

The Delphi method is a systematic way of determining expert consensus among a variety of key stakeholders and a common approach in mental health research and priority-setting exercises (Hasson et al., 2000). It uses an iterative multi-staged approach to transform opinion into group consensus on a given topic. The Delphi method is ideal to use when there are multiple stakeholder groups as all perspectives are weighted evenly. It does not require representativeness, rather the focus is on recruiting stakeholders with a diversity of expertise (Jorm, 2015). The validity of the Delphi method is supported by research on the ‘wisdom of crowds’, which investigates the conditions under which groups (‘crowds’) produce optimal decisions (Jorm, 2015, 2025). This research shows that groups make better quality decisions when they are selected for relevant expertise, involve cognitive diversity, make independent judgements, and have opportunity for sharing their expertise and judgements through anonymous feedback (Jorm, 2025). The Delphi methodology adopted here met all these criteria.

The value-weighting exercise, a form of resource allocation, was used to capture the breadth and difference of opinion of research priorities and to refine the endorsed research topics to those of the highest research priority (Paul et al., 2011). This method allows participants to anonymously indicate the perceived value of each research topic applied in the context of a limited funding base.

Ethics approval was granted by the Human Research Ethics Committee of the University of Melbourne (project no: 27549).

### Participants

#### Inclusion criteria

We recruited stakeholders into two panels according to their expertise: (1) Lived Experience expertise (parents and carers of children) and (2) Professional expertise. These stakeholder groups have different sources of expertise on the topic, and research shows that such cognitive diversity is likely to result in better quality judgements (Jorm, 2025). Furthermore, forming separate panels meant that both forms of expertise were given equal weight. Panellists were also required to be living in Australia, aged 18 years or above and could read and write English.

Panellists were eligible to participate in the Lived Experience panel if they had experience supporting a child below 12 years with a mental health problem and were involved as Lived Experience advocates or carer representatives.

For the Professional panel, a diverse range of expertise was eligible, including clinicians, service providers, researchers, educators, policymakers and any other professionals with experience in supporting or promoting child mental health. Clinicians needed to have at least 2 years of experience in child mental health and be registered to practice. Researchers needed to have published peer-reviewed research in the past 10 years related to child mental health trials. Educators were required to have at least 2 years of experience in child mental health in an educational setting. Other expertise was considered on a case-by-case basis for roles related to child wellbeing and mental health such as service providers and policymakers.

At the time of recruitment, three panels were proposed: Lived Experience; Researchers & Clinicians; and Other Expertise. However, due to recruitment difficulties, the Other Expertise panel was merged with the Researcher & Clinician panel and renamed the Professional panel. Where appropriate, some members of the Other Expertise panel were moved into the Lived Experience panel if they met the criteria for this panel.

#### Project reference group

A Project Reference Group was established comprising a diverse range of stakeholders to provide expert guidance throughout the project. The group included representative members nominated from GMA’s advisory committees (Community Engagement, Scientific, Early-Mid Career Network, and the Core Methods health economist group) as well as a mental health practitioner, an Aboriginal person working with communities in child protection, and a Lived Experience representative.

#### Recruitment

Recruitment flyers were advertised via GMA’s membership networks, and other relevant organisations in child mental health through social media channels, newsletters and direct callouts via email. Subject matter experts were also approached directly via email if they were a corresponding author on any of the studies included in our review detailed above, or via the Project Reference Group’s professional contacts. Eligible participants also provided recommendations or passed on the study information directly.

Interested participants completed a brief expression of interest survey online to allow the research team to assess eligibility and relevance of expertise against the inclusion criteria. If panellists had more than one area of expertise, they selected the panel that they preferred based on their most recent or in-depth experience. Additional recruitment occurred for Phase 2 (resource allocation exercise) to account for attrition and to seek input from any key stakeholder groups that were not included in Rounds 1–3.

Participants with Lived Experience were offered an e-gift card as a reimbursement for their time at the end of the final survey.

### Development of the Delphi survey

#### Identification of potential priorities

An important first step in consensus-based research is the synthesis of relevant information to highlight research gaps (Gattrell et al., 2024). To inform the present research prioritisation study, a rapid review was conducted by the research team to map the range and extent of clinical trials conducted in Australia on child mental health interventions in the last 10 years (Tsiamis, 2024). Clinical trials were defined as ‘. . . *any research study that prospectively assigns human participants or groups of humans to one or more health-related interventions to evaluate the effects on health outcomes*’ (World Health Organisation, 2020). Child mental health problems were defined using the *Diagnostic and Statistical Mental of Mental Disorders, Fifth Edition* (*DSM*-5) criteria (American Psychiatric Association, 2022) or the *Diagnostic Classification of Mental Health and Developmental Disorders of Infancy and Early Childhood: 0-5* (DC:0-5) (ZERO TO THREE, 2016). Child mental health problems included mental health disorders as well as symptoms. Data were extracted on each study’s aims, trial design, participant characteristics, type of mental health problem, intervention details, funding source, and child outcome measures, and this taxonomy formed the foundation of the first round of the Delphi survey.

In addition to publications, we examined targeted calls for research grants, and priority-setting policy documents for emerging research areas that could be potential priorities from the year 2013 to May 2023. Content analysis identified additional research priorities that had not been identified in the rapid review.

#### Development of the Delphi survey

The authors developed the framework of the Delphi survey based on the content analysis. The authors worked closely with the Project Reference Group who were involved in defining the project research question, defining key inclusion criteria and definitions, providing feedback on the survey design, proposing additional priority topics not yet considered, and pilot testing the Round 1 survey. A list of the 30 research topics presented in the survey are displayed in Supplementary Table 1. Most topics had multiple sub-items for rating.

## Procedure

### Phase 1 – Delphi consensus study (Rounds 1−3)

The Round 1 Delphi online survey was sent to eligible panel members in October 2023. Panellists were encouraged to complete the survey within 2 weeks, and the survey was closed in December 2023 once a suitable sample size of at least 30 people per panel was reached.

**Figure 1.**
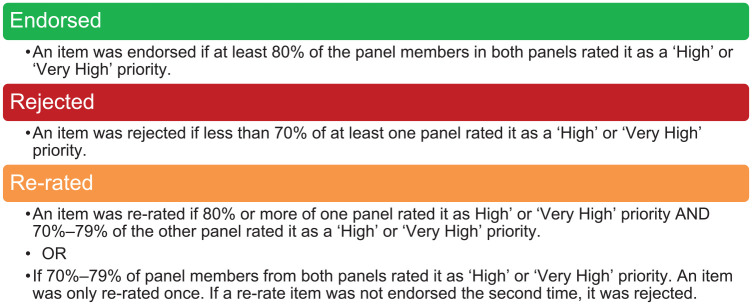
Criteria for analysing data for endorsement, re-rating or rejection.

Panellists were asked to rate the level of priority for specific topics and sub-items on a 5-point Likert-type scale – ‘very low priority’ (1), ‘low priority’ (2), ‘medium priority’ (3), ‘high priority’ (4), and ‘very high priority’ (5). There was also the opportunity to suggest new items in Round 1 for rating in Round 2. To support the panellist’s rating of priorities, information was provided on prevalence and burden data from the Global Burden of Disease 2019 database, as well as a summary of research effort for that topic identified in the review. This highlighted areas where there was a higher research effort or gaps in terms of the type of mental health problem, the type or spectrum of intervention, or specific population or age groups. At the end of the Round 1 survey, panellists were asked about the extent to which gaps in research effort, societal cost, burden on the child, or prevalence of the mental health problem informed their ratings. See Figure 1 for the criteria for item endorsement, re-rating or rejection.

At the end of each round, panellists were sent a report outlining the results. In Rounds 2 and 3, items to be re-rated were displayed with the percentage of each panel’s response so that participants could compare their responses to each panel’s ratings. This was to aid participants’ decision-making as to whether they modify or maintain their ratings in the subsequent round.

The authors reviewed all qualitative feedback from panellists and developed new items to be included for rating in Round 2. The Project Reference Group reviewed and endorsed the new topics and sub-items developed by the working group. Round 3 included re-rating of new topics and sub-items proposed by panellists that did not meet consensus in Round 2.

### Phase 2 – Resource allocation exercise

After the third Delphi round concluded, a fourth survey was conducted to refine the list of endorsed priorities to those of the highest priority using a resource allocation exercise. The list of items for the resource allocation exercise included endorsed items in Rounds 1−3 where 85% or more of each panel rated it as a high or very high priority. Panellists were asked how they would allocate 100 points of funding across research priorities in three areas (promotion trials, prevention and assessment trials and treatment trials).

Original panel members from the Delphi study and new participants were invited to account for areas of expertise that were not represented in Rounds 1−3 as well as attrition. To preserve the equal weighting of both Lived Experience and Professional expert panels, we calculated the mean allocation of points for each item by panel and then calculated the mean of both panels. Items were determined as a top research priority in the final round if the mean was above the expected amount if each priority was equal (i.e. the allocation of 100 points divided by the number of items being rated).

## Results

### Panellists

Seventy-five eligible participants were sent the Round 1 survey, 88.0% (*n* = 66) of whom completed Round 1, 27 on the Lived Experience panel and 39 on the Professional panel. The retention rate was high for both panels, with 93.6% of Lived Experience panellists and 79.5% of Professional panellists completing Round 3 (see Table 1). Sixty-eight participants completed the fourth survey, 16 of whom were new participants recruited to Phase 2 (2 new Lived Experience and 16 new Professional participants including targeted recruitment of child psychiatrists).

**Table 1.** Participant survey completion and retention.

Round	Participants	
	Lived Experience Panel *N* (%)	Professional Panel *N* (%)
Round 1 completed	27	39
Round 2 completed (retention)	26 (96.3%)	35 (89.7%)
Round 3 completed (retention)	25 (92.6%)	31 (79.5%)

The Delphi panellists’ demographics and areas of expertise can be found in Table 2. The Professional panel included a mix of expertise including researchers, clinicians, policymakers and other experts. Many panellists reported multiple sources of expertise, that is, a clinician and researcher, or a policymaker and researcher.

**Table 2.** Panellist demographic and area of expertise information.

	Phase 1–Round 1 (*n* = 66)		Phase 2–Round 4^a^ (*n* = 68)	
	Mean (SD)	Range	Mean (SD)	Range
Age	44.6 (11.0)	18−65 years	44.7 (11.1)	18−67 years
	N	%	N	%
Gender				
Woman	57	86.4	60	88.2
Man	9	13.6	12	17.6
Non-binary	0	0.0	0	0.0
State				
Victoria	36	54.5	39	57.4
New South Wales	16	24.2	18	26.5
Queensland	10	15.2	10	14.7
South Australia	2	3	1	1.5
Australian Capital Territory	1	1.5	1	1.5
Western Australia	1	1.5	1	1.5
Northern Territory	0	0.0	1	1.5
Tasmania	0	0.0	1	1.5
**Mental health problem(s) experience^b^**				
Anxiety	24	36.4	29	42.6
Attention-deficit/hyperactivity disorder (ADHD)	19	28.8	23	33.8
Autism spectrum disorder	19	28.8	20	29.4
Trauma (Trauma- and Stressor-related disorders)	10	15.2	14	20.6
Mood (depression, bipolar)	9	13.6	13	19.1
Conduct & externalising behaviours	8	12.1	12	17.6
Eating and feeding disorders	6	9.1	10	14.7
Obsessive-compulsive disorder	4	6.1	4	5.9
Self-harm	2	3.0	2	2.9
Sleep problems	2	3.0	4	5.9
Intellectual disability	2	3.0	5	7.4
Attachment disorder/problems	2	3.0	4	5.9
Learning disorder	1	1.5	4	5.9
Other	6	9.9	6	8.8
No specific mental health problem reported	17	25.8	23	33.8
**Professional Panel discipline^b^**	(Professional panel = 39)		(Professional panel = 45)	
Researcher/Academic	25	64.1	29	69.0
Clinician	15	38.5	22	52.4
Policymakers	4	10.3	**3**	7.1
Service Provider	2	5.1	1	2.4
Educator	1	2.6	1	2.4
Other expertise	2	5.1	2	4.8
**Professional panel child age expertise^b^**				
Primary school (5−12 years)	28	71.8	28	62.2
Preschool (3−5 years)	23	59.0	21	46.7
Toddler (1−3 years)	16	41.0	14	31.1
Antenatal or Infant (up to 1 year)	8	20.5	7	15.6
Not reported	9	23.1	4	8.9
**Clinician profession**				
Psychologist	8	53.3	14	51.9
Paediatrician	3	20.0	4	14.8
Occupational therapist	1	6.7	2	7.4
GP	1	6.7	1	3.7
Nurse	1	6.7	1	3.7
Other registration (Counsellor)	1	6.7	1	3.7
Psychiatrist	0	0.0	4	14.8
Total clinicians	15		27	

a Additional recruitment was undertaken for Round 4.

b Categories are not mutually exclusive.

### Phase 1 – Delphi study (Rounds 1−3)

**Figure 2.**
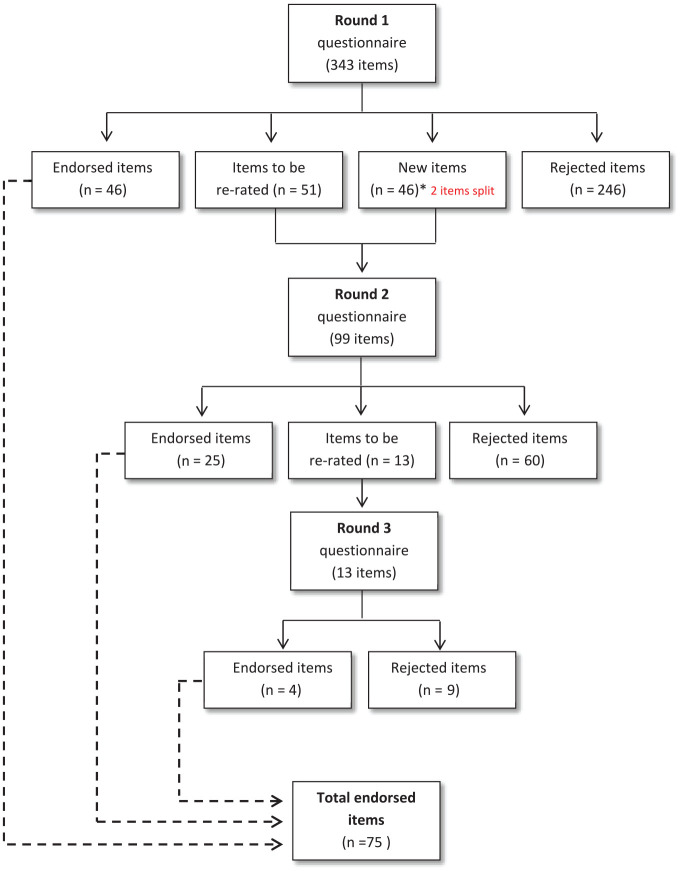
Summary of item ratings and Delphi Rounds 1−3 results.

Round 1 contained 343 items across 30 topics for rating spanning five sections (High-level categories of trials, Promotion, Prevention, Treatment and Assessment). In the Round 2 survey, 51 items were re-rated across all five sections, in addition to rating 46 new items, and two items split into two (see Figure 2). In Round 3, 13 items were re-rated.

After three rounds, **75 items were endorsed as research priorities** out of 391 potential topics (19.2%) (see Table 3 for a summary of all endorsed and rejected items). Fourteen of these were new items proposed by panellists in Round 1. Prevention trials had the highest number of endorsed topics (*n* = 28/72, 38.9%). This was followed by high-level categories of trials (*n* = 16/54, 29.6%), treatment trials (*n* = 16/146, 11.0%), promotion trials (*n* = 10/27, 37.0%), and finally assessment trials (*n* = 5/91, 5.5%). See Supplementary Tables 2 and 3 for a full list of the endorsed 75 priorities and the panellists ratings of all sub-items.

**Table 3.** Summary of endorsed and rejected research priorities by section and research topic (Delphi Round 3).

**Section 1: High-level categories**
**Q1. Child age and developmental stage** ✔: Preschool (3−4.9 years); Early primary school (5−8.9 years); Late primary school (9−12 years) ✖: Infant (0−1 years); Toddler (1−2.9 years)
**Q2. Child gender** ✔: Gender diverse (trans or non-binary) ✖: Boy; Girl
**Q3. Priority populations** ✔: Aboriginal and/or Torres Strait Islander peoples; Culturally and Linguistically Diverse; Rural or regional areas; *Mental health problems in children with neurodevelopmental disorders ✖: LGBTQIA+
**Q4. Spectrum of intervention** ✔: Prevention; Treatment ✖: Promotion; Assessment
**Q5. Intervention participants** ✔: Children; Parents and caregivers ✖: Families (including kin and other non-primary caregivers); Educators; Health professionals; Other adults in the community (e.g. coaches, religious leaders)
**Q6. Type of mental health problem** ✔: Anxiety disorders; *Co-occurring mental health problems ✖: Attention Deficit/Hyperactivity Disorder (ADHD); Autism spectrum disorder; Communication disorders (speech or language disorders); Disruptive, impulse-control and conduct disorders; Dissociative disorders; Elimination disorders; Excessive crying disorders; Feeding and eating disorders; Intellectual Disabilities; Mood disorders (Bipolar, Depression); Motor disorders (e.g. developmental coordination disorder or Tourette’s); Obsessive-compulsive disorders (OCD); Schizophrenia Spectrum & Other Psychotic Disorders; Self-harm; Sleep-wake or sleep disorders; Somatic symptom−related disorders; Specific learning disorder; Substance-related and addictive disorders (including vaping); Trauma-and stressor-related disorders (e.g. PTSD); *Other neurodevelopmental disorders associated with prenatal alcohol exposure
**Q7. Other aspects of trial design** ✔: Real-world effectiveness trials – Evaluation of interventions in settings that reflect real-world practice; *Consideration of people’s past experience of trauma ✖: Trials that prioritise the voice of lived experience (children and parents/caregivers); Co-designing interventions with the population groups where the intervention will be trialled and implemented; Larger multicentre trials; Testing effects of implementation strategies (such as uptake) in practice; Trials that evaluate integration and collaboration between sectors and disciplines; *Consistent outcome measures for a range of domains, e.g. psychological distress, sleep, parent-child relationship, quality of life
**Section 2: Promotion trials**
**Q8. Reducing stigma related to child mental health problems in specific participant groups** ✔: Parents and caregivers; Educators ✖: Children (towards other children); Families (including kin and other non-primary caregivers); Health professionals; Other adults in the community (e.g. coaches, religious leaders)
**Q9. Improving mental health literacy for supporting children in specific participant groups** ✔: Parents and caregivers; Educators ✖: All other groups rejected. See Q5 for a full list.
**Q10. Improving the wellbeing of children experiencing mental health problems** ✔: Endorsed. Nil sub-items
New Q. *Improving the wellbeing of children experiencing mental health problems ✖: Rejected. Nil sub-items
New Q. *Improving the wellbeing of parents/caregivers of children experiencing mental health problems ✔: Endorsed. Nil sub-items
**Q11. Promotion trials by priority group** ✔: Aboriginal and/or Torres Strait Islander peoples; *Mental health problems in children with neurodevelopmental disorders ✖: Culturally and Linguistically Diverse; Rural or regional areas; LGBTQIA+
**Q12. Setting of promotion trials** ✔: School/early childhood education ✖: Community; Home – individual or family-based intervention; Online/digital
**Q13. Other aspects of promotion trial design** ✔: Long-term follow-up for promotion trials
**Section 3: Prevention trials**
**Q14. Type of prevention** ✔: Indicated prevention ✖: Universal prevention; Selective prevention
**Q15. Age of prevention** ✔: Preschool (3−4.9 years); Early primary school (5−8.9 years); Late primary school (9−12 years); Transition points (i.e. from preschool to primary/ primary to secondary school) ✖: Prenatal period or infancy (up to 1 year); Toddler (1−2.9 years)
**Q16. Setting of prevention trials** ✔: School/early childhood education; Home – individual or family-based intervention; ✖: All other sub-items rejected. See Q 12 for full list
**Q17. Prevention in specific participant groups** ✔: Children; Parents and caregivers; Educators ✖: All other groups rejected. See Q5 for a full list.
**Q18. Children who have adverse experiences or are in at risk groups for (selective) prevention** ✔: Children who experience child abuse; Children who have been in contact with (or at risk of) the justice system; Children in out-of-home care / state care or subject to notifications of abuse; *Children experiencing multiple adversities ✖: Children of parents with a mental illness; Children of parents with substance use issues; Children placed in care with kin/community networks; Children with chronic illness(es) and/or comorbidities (physical); Children with disabilities; Children who experience bullying
**Q19. Prevention trials by priority group** ✔: Aboriginal and/or Torres Strait Islander peoples; Culturally and Linguistically Diverse; Rural or regional areas; ✖: LGBTQIA+; *Mental health problems in children with neurodevelopmental disorders
**Q20. Type of mental health problem for prevention** ✔: Anxiety disorders; Trauma-and stressor-related disorders (e.g. PTSD); *Self-harm and injury (non-suicidal behaviour) ✖: All other mental health problems were rejected. See Q6 above for a full list of items or Supplementary 3.
**Q21. Type of intervention for prevention** ✔: Psychological or psychosocial; *Interventions targeting the social determinants of health ✖: Pharmacological (i.e. medication); Complementary medicines; Lifestyle interventions; Devices
**Q22. Other aspects of prevention trial design** ✔: Understanding what works for whom; Long-term follow-up for prevention trials ✖: Understanding the intervention components that have a positive impact (active ingredients, key mechanisms or mediators); Optimising existing prevention interventions (e.g. optimal dose/length, implementation); Side-effects/negative effects of prevention interventions; Trials focused on health economics of treatment – e.g. cost-effectiveness and cost-utility; Transdiagnostic approach to symptoms and co-occurring mental health disorders; Implementation of existing prevention interventions
**Section 4: Treatment trials**
**Q23. Setting of treatment trials** ✔: School/early childhood education; Home – individual or family-based intervention ✖: All other sub-items rejected. See Q 12 for full list
**Q24. Type of mental health problem for treatment** ✔: Anxiety disorders; Trauma-and stressor-related disorders (e.g. PTSD); *Treatment for co-occurring mental health problems; *Self-harm and injury (non-suicidal behaviour); *Suicide including thoughts and attempts ✖: All other mental health problems were rejected. See Q6 above for a full list of items or Supplementary 3.
**Q25. Type of intervention for treatment** ✔: Psychological or psychosocial ✖: Pharmacological (i.e. medication); Complementary medicines; Lifestyle interventions; Devices
**Q26. Treatment intervention type for specific mental health problems** ✔: Psychological or psychosocial interventions for anxiety problems; Psychological or psychosocial interventions for mood disorders; Psychological or psychosocial interventions for self-harm; Psychological or psychosocial interventions for Trauma-and stressor-related disorders (e.g. PTSD) ✖: All other items rejected. See Supplementary Table 3 for all items.
**Q27. Other aspects of treatment trial design**✔: Understanding the intervention components that have a positive impact (active ingredients, key mechanisms or mediators); Understanding what works for whom; Transdiagnostic approach to symptoms and co-occurring mental health disorders; Long-term follow-up for treatment trials; *Transdiagnostic approach to symptoms and co-occurring mental health disorders ✖: Interventions for non-responders; Optimising existing treatment interventions (e.g. optimal dose/length, implementation); Side-effects/negative effects of prevention interventions; Trials focused on health economics of treatment – e.g. cost-effectiveness and cost-utility; Implementation of existing treatment interventions; *Comparison of intervention types, e.g. pharmacological vs lifestyle vs psychological interventions
**Section 5: Assessment trials**
**Q28. Screening and diagnostic tools** ✔: Improved screening tools (0−12 years) and in *younger children (0−8.9 years); *Improved diagnostic tools for mental health problems in children with neurodevelopmental disorders ✖: Improved diagnostic tools for specific disorders; Other assessment tools relevant to mental health promotion e.g. mental health literacy/stigma; *Improved diagnostic tools for co-occurring mental health disorders; *Assessment of strengths
**Q29. Evaluating the impact of assessing child mental health indicators at routine developmental checks** ✔: Endorsed. Nil sub-items
**Q30. What assessment tools are a priority for which mental health problems** ✖: All sub-items rejected.
**New Q. Trials evaluating linking screening with appropriate care** ✔: Endorsed. Nil sub-items
**New Q. Assessment of strengths by mental health problem** ✖: All sub-items rejected

* Indicates new items suggested by panellists. Please refer to Supplementary Table 3 for a full list of all items and their ratings.

Mental health problems endorsed as research priorities for prevention and treatment trials included anxiety disorders, trauma-and stressor-related disorders (e.g. post-traumatic stress disorder [PTSD]), self-harm and injury (non-suicidal behaviour). In addition, suicide (including thoughts and attempts) and co-occurring mental health problems were endorsed as a priority for treatment trials only. While a range of intervention types were presented for rating, only psychological and psychosocial interventions were endorsed as a priority. Panellists agreed that school or early education and home settings, with a focus on parents/caregivers and educators in these settings, were a priority for prevention and treatment trials. Promotion trials aimed at reducing stigma and improving mental health literacy in parents/caregivers and educators in school or early education settings were priorities.

Aspects of trials regardless of the mental health problem or type of treatment were also endorsed, including further investigation on how prevention or treatment interventions work, who they work for, and whether they work in real-world settings. Long-term follow up of trials was also seen as a common priority for prevention, promotion, and treatment trials.

When considering the assessment of child mental health problems, improved screening and diagnostic tools were endorsed as a research priority across all mental health problems in neurodiverse children as well as for neurotypical children.

**Figure 3.**
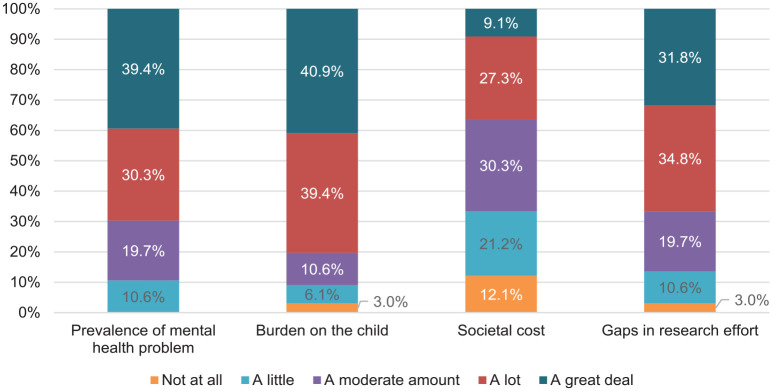
Summary of what informed participant ratings in the Delphi study.

The extent to which gaps in research effort, societal cost, burden on the child, or prevalence of the mental health problem informed participant ratings is illustrated in Figure 3.

#### Differences between panels

Although the focus of this study was to identify priorities that had consensus across key stakeholders, we observed some differences between panels. The Lived Experience panel endorsed 36.3% of items compared to 19.4% for the Professional panel. There were 67 items across Rounds 1−3 that were endorsed by the Lived Experience panel but rejected by the Professional panel. In contrast, there was only one item that was endorsed by the Professional panel but rejected by the Lived Experience panel (‘*Other aspects of treatment trial design – Implementation of existing treatment interventions*’). Due to these items not reaching consensus by both panels, they were rejected. One item had 100% endorsement by the Lived Experience panel but was not included in the list of 75 research priorities as it was not endorsed at a high enough rate by the Professional panel (‘*Trials that prioritise the voice of lived experience of children and parents/caregivers*’). Despite the difference between the panels in the percentage of items endorsed, the Pearson’s correlation between the total endorsement percentage across the 391 potential priority items between the Lived Experience panel and the Professional panel was high (*r* = 0.66).

### Phase 2 – Resource Allocation (Round 4)

At the end of Round 3, 38 items were endorsed as a higher priority, where 85% or more of both panels rated them as either a ‘High’ or ‘Very High’ priority (Supplementary Table 4). Of these 38 items, **12 priorities were identified as the top research priorities for child mental health intervention trials** in the final phase of this study. These included two promotion trial priorities, seven prevention and assessment trial priorities, and four treatment trial priorities.

The top research priorities are presented in Table 4. Supplementary Tables 5.1 and 5.2 present all items by ranking and include a comparison of results for the Lived Experience panel and Professional panel.

**Table 4.** Summary of the top research priorities for child mental health (results from Round 4).

Research Priority
**Promotion trials:** ● Improving the wellbeing of children experiencing mental health problems ● Improving mental health literacy in parents/caregivers
**Prevention and Assessment trials:** ● Selective prevention in at risk population groups for one of the following adverse childhood events: *Children who experience child abuse; children in out-of-home care/state care or subject to notifications of abuse; children in contact with (or at risk of) the justice system; children with disabilities; or children experiencing multiple adversities.* ● Age – Prevention in early primary school age (5−8.9 years) ● Age – Prevention in late primary school age (9−12 years) ● Age – Prevention at transition points (i.e. from preschool to primary school, or primary to secondary school) ● Prevention setting – Home: individual or family-based intervention ● Evaluating the impact of assessing child mental health indicators at routine developmental checks (early intervention & prevention) ● Other aspects of prevention trial design – Real-world effectiveness prevention trials: Evaluation of interventions in settings that reflect real-world practice
**Treatment trials:** ● Treatment of co-occurring mental health problems ● Other aspects of treatment trial design – Real-world effectiveness treatment trials: Evaluation of interventions in settings that reflect real-world practice ● Other aspects of treatment trial design – Understanding the intervention components that have a positive impact (active ingredients, key mechanisms or mediators) ● Other aspects of treatment trial design – Understanding what works for whom

## Discussion

This study included a multi-phased approach to identify the research priorities for future clinical trials focused on child mental health interventions in Australia. Lived Experience and Professional panellists endorsed 75 research priorities in a Delphi study across the spectrum of intervention (i.e. promotion, prevention, assessment, treatment) and high-level categories applicable to all types of trials. The final resource allocation exercise refined these 75 research topics to 12 top research priorities for the GMA CTN and other key stakeholders involved in child mental health trials. These include two promotion trial priorities, seven prevention and assessment trial priorities, and four treatment trial priorities. Population groups that are a top priority include those at risk of ACEs and the primary school age groups (5−12 years) including transition points around this time. A focus on promotion to improve the wellbeing of children with mental health problems and the treatment of children with co-occurring mental health problems were also identified as top priorities, as well as aspects of treatment trials that apply regardless of the mental health problem or type of treatment (how treatments work, who they work for, and whether they work in real-world settings).

Findings suggest that the top 12 research priorities were those that avoided picking ‘winners’ and ‘losers’ among specific mental health problems, priority populations or types of intervention, but were aspects of clinical trials that could be adopted widely. For instance, there were few specific mental health problems identified as priorities, and none that were judged as a top research priority. Rather, a top priority was clinical trials focusing on treatment of co-occurring mental health problems. Similarly, there were no priority populations (such as Aboriginal and/or Torres Strait Islander peoples) that reached the top 12 priorities. Furthermore, no treatment or prevention intervention types were endorsed as top priorities, but there was consensus on evaluating interventions in settings that reflect real-world practice. This may reflect the relatively high proportion of Australian clinical trials conducted in university clinics rather than community or health service settings (Tsiamis, 2024). In addition, identifying moderators and mediators of treatments was a top priority, reflecting the call for a more nuanced understanding of how treatments work and in what circumstances (Kazdin, 2007). The top 12 research priorities are likely to be helpful to the Australian mental health research sector, since it means that they are applicable to all researchers, irrespective of whether one’s research area focuses on a particular intervention type or mental health problem.

Despite this, for prevention trials, there was an emphasis on selective prevention, rather than universal or indicated prevention, and on the family home as a top priority setting rather than schools, community, or health settings. School-based universal prevention interventions, which are often delivered as digital programmes, have been popular for the adolescent period (Birrell et al., 2025), but our panellists rejected universal prevention and the digital setting as priorities during childhood. Instead, there was a focus on selective prevention in children experiencing ACEs. It is estimated nearly three-quarters of Australian children have been exposed to at least one ACE, and these estimates are likely higher in vulnerable communities (Zubrick et al., 2005). It is well-recognised that prevention and early intervention focused on children experiencing ACEs can reduce the impact on mental health outcomes experienced in childhood and later in life (Grummitt et al., 2024). This is reflected throughout the National Child’s Mental Health and Wellbeing Strategy, with a call to focus on recovery and healing from stress and trauma (National Mental Health Commission, 2021).

For promotion trials, the top research priorities were improving the wellbeing of children experiencing mental health problems and improving the mental health literacy of parents/caregivers. These align with government policies (Department of Health and Aged Care [DoHAC], 2019; National Mental Health Commission, 2021) and another priority study (Sharma et al., 2021), and reflect the limited research on promotion in children and parents/caregivers (Johnson et al., 2023; Tsiamis, 2024). These findings support calls for greater investment in increasing community levels of mental health literacy for childhood mental health disorders (Tully et al., 2019), particularly in parents and caregivers, as well as educators. A priorities study looking at improving child and youth mental health in Australia and New Zealand echoes many of the findings in this study and reports *education settings* as key in *supporting the wellbeing of, and preventing mental health problems in children and young people*, as well as a need to *reduce stigma* (Sharma et al., 2021).

Other research have identified community-based interventions (Novins et al., 2021; Sahle et al., 2021), community service models (DoHAC, 2023; Taylor et al., 2022), and lifestyle interventions such as sleep, physical activity, and screen time (Taylor et al., 2022) as a priority for future research for both mental health and ACEs. However, our findings diverged from these priorities as lifestyle interventions or interventions delivered in community settings were rejected. Only psychological and psychosocial interventions were endorsed as priorities, despite these intervention types having the highest research effort in our rapid review (Tsiamis, 2024). Clinical practice guidelines frequently cite physical activity, sleep, nutrition, and other lifestyle interventions as interventions for paediatric depression and other child mental health problems (Biddle and Asare, 2011; Campisi et al., 2021). While the need to consider and improve lifestyle factors for the prevention and treatment of mental health problems is widely acknowledged, changing behaviour and increasing uptake are an ongoing challenge in intervention research.

The Delphi method is a rigorous approach to determining consensus on a topic. Nevertheless, there are many decisions throughout the process that may influence the outcome. For a research prioritisation process, the biggest challenge is in drafting a questionnaire of priorities that is comprehensive but feasible to complete by panellists. It was not feasible to include every permutation of research area (e.g. home-based psychological treatment of anxiety disorders in primary-school-aged boys in rural/regional areas), so we focused on higher level categories with some exceptions. We cannot rule out that a differently structured questionnaire would have identified different priorities. However, this is mitigated by the opportunity for panellists to provide feedback and suggest new items for consideration. Another important decision was in the resource allocation round, where we deliberately chose to ask participants to rate priorities within sections (promotion, prevention, treatment) rather than across all the endorsed priorities. This was because we recognised the difficulty in judging importance across different categories, but it meant that the top priorities may have been different if the sections were not treated independently.

### Strengths and limitations

The study’s strengths include the high retention rate in the Delphi study, the diversity of expertise, the breadth of research topics covered, and the rapid review’s supporting information on research effort that was provided to panellists to inform their ratings.

Evidence shows the cognitive diversity of groups improves the quality of decisions (Jorm, 2015). By keeping the Lived Experience panel separate from the Professional panel and requiring a high level of endorsement from both, we have given equal weight to the expertise and values of each group. Although the Lived Experience panel endorsed a higher percentage of items than the Professional panel, there was overall a high degree of concordance in the ordering of their priorities. Recruiting panellists with heterogeneous experience across mental health problems and age groups also added to the diversity of expertise in the study.

Alongside these strengths, some limitations should be noted. While the study included a diverse sample of experts with a range of experience and expertise across disciplines and professions, there was limited participation from social workers, nurses, GPs, occupational therapists, and those with expertise in communication disorders.

## Conclusion

Study findings will inform the GMA CTN’s review, prioritisation, and endorsement of future clinical trials in child mental health. The findings can also be used to inform other key funders of Australian mental health research and other key stakeholders on where clinical trial research effort has focused in the past and what should be considered a priority in the future.

## Supplemental Material

sj-docx-1-anp-10.1177_00048674251345318 – Supplemental material for Setting Australian research priorities for child mental health clinical trials: A Delphi study Supplemental material, sj-docx-1-anp-10.1177_00048674251345318 for Setting Australian research priorities for child mental health clinical trials: A Delphi study by Ellie Tsiamis, Anthony Jorm, Lakshmi Neelakantan and Amy Morgan in Australian & New Zealand Journal of Psychiatry

sj-xlsx-2-anp-10.1177_00048674251345318 – Supplemental material for Setting Australian research priorities for child mental health clinical trials: A Delphi study Supplemental material, sj-xlsx-2-anp-10.1177_00048674251345318 for Setting Australian research priorities for child mental health clinical trials: A Delphi study by Ellie Tsiamis, Anthony Jorm, Lakshmi Neelakantan and Amy Morgan in Australian & New Zealand Journal of Psychiatry

sj-xlsx-3-anp-10.1177_00048674251345318 – Supplemental material for Setting Australian research priorities for child mental health clinical trials: A Delphi study Supplemental material, sj-xlsx-3-anp-10.1177_00048674251345318 for Setting Australian research priorities for child mental health clinical trials: A Delphi study by Ellie Tsiamis, Anthony Jorm, Lakshmi Neelakantan and Amy Morgan in Australian & New Zealand Journal of Psychiatry
