# Supplementary material for: Setting Australian research priorities for child mental health clinical trials: A Delphi study
Source: Aust N Z J Psychiatry. 2025 Jun 24;59(8):740–53. doi: 10.1177/00048674251345318 (PMC12280242; doi:10.1177/00048674251345318)
Supplement: sj-docx-1-anp-10.1177_00048674251345318 – Supplemental material for Setting Australian research priorities for child mental health clinical trials: A Delphi study [file sj-docx-1-anp-10.1177_00048674251345318.docx]

# Supplementary tables

**Supplementary Table 1. Potential research priority topics and sub items presented for rating in Round 1.**

| **Topic** | **Items for rating** |
| --- | --- |
| **Section 1: High-level categories** | |
| Q1. Child age and developmental stage | Infant (0-1 years); Toddler (1-2.9 years); Preschool (3-4.9 years); Early primary school (5-8.9 years); Late primary school (9-12 years); |
| Q2. Child gender | Boy; Girl; Gender diverse (trans or non-binary) |
| Q3. Priority populations | Aboriginal and/or Torres Strait Islander peoples; Culturally and Linguistically Diverse; Rural or regional areas; LGBTQIA+ |
| Q4. Spectrum of intervention | Promotion; Prevention; Assessment; Treatment |
| Q5. Intervention participants | Children; Parents and caregivers; Families (including kin and other non-primary caregivers); Educators; Health professionals; Other adults in the community (e.g. coaches, religious leaders) |
| Q6. Type of mental health problem | Anxiety disorders; Attention Deficit/Hyperactivity Disorder (ADHD); Autism spectrum disorder; Communication disorders (speech or language disorders); Disruptive, impulse-control and conduct disorders; Dissociative disorders; Elimination disorders; Excessive crying disorders; Feeding and eating disorders; Intellectual Disabilities; Mood disorders (Bipolar, Depression); Motor disorders (e.g. developmental coordination disorder or Tourette's); Obsessive-compulsive disorders (OCD); Schizophrenia Spectrum & Other Psychotic Disorders; Self-harm; Sleep-wake or sleep disorders; Somatic symptom related disorders; Specific learning disorder; Substance-related and addictive disorders (including vaping); Trauma-and stressor-related disorders (e.g. PTSD) |
| Q7. Other aspects of trial design | Trials that prioritise the voice of lived experience (children and parents/caregivers); Co-designing interventions with the population groups where the intervention will be trialled and implemented; Real-world effectiveness trials - Evaluation of interventions in settings that reflect real-world practice; Larger multi-centre trials; Testing effects of implementation strategies (such as uptake) in practice; Trials that evaluate integration and collaboration between sectors and disciplines, e.g. between the education and health service system. |
| **Section 2: Promotion trials** | |
| Q8. Reducing stigma related to child mental health problems in specific participant groups | Children (towards other children); Parents and caregivers; Families (including kin and other non-primary caregivers); Educators; Health professionals; Other adults in the community (e.g. coaches, religious leaders) |
| Q9. Improving mental health literacy for supporting children in specific participant groups | Children; Parents and caregivers; Families (including kin and other non-primary caregivers); Educators; Health professionals; Other adults in the community (e.g. coaches, religious leaders) |
| Q10. Improving the wellbeing of children experiencing mental health problems | Nil sub items for rating |
| Q11. Promotion trials by priority group | Aboriginal and/or Torres Strait Islander peoples; Culturally and Linguistically Diverse; Rural or regional areas; LGBTQIA+ |
| Q12. Setting of promotion trials | School/early childhood education; Community (e.g. sporting clubs, music or arts programs, play groups, churches); Health service; University (includes university-based clinics); Home – individual or family-based intervention; Online/digital |
| Q13. Other aspects of promotion trial design | Long term follow up for promotion trials (i.e. measuring if there is long-term improvement in mental health outcomes in children) |
| **Section 3: Prevention trials** | |
| Q14. Type of prevention (universal, selective, indicated) | Universal prevention; Selective prevention; Indicated prevention |
| Q15. Age of prevention | Prenatal period or infancy (up to 1 year); Toddler (1-2.9 years); Preschool (3-4.9 years); Early primary school (5-8.9 years); Late primary school (9-12 years); Transition points (i.e. from pre-school to primary/ primary to secondary school) |
| Q16. Setting of prevention trials | School/early childhood education; Community (e.g. sporting clubs, music or arts programs, play groups, churches); Health service; University (includes university-based clinics); Home – individual or family-based intervention; Online/digital |
| Q17. Prevention in specific participant groups | Children; Parents and caregivers; Families (including kin and other non-primary caregivers); Educators; Health professionals; Other adults in the community (e.g. coaches, religious leaders) |
| Q18. Children who have adverse experiences or are in at risk groups for (selective) prevention | Children of parents with a mental illness; Children of parents with substance use issues ; Children who experience child abuse; Children who have been in contact with (or at risk of) the justice system; Children in out-of-home care / state care or subject to notifications of abuse; Children placed in care with kin/community networks; Children with chronic illness(es) and/or comorbidities (physical); Children with disabilities; Children who experience bullying |
| Q19. Prevention trials by priority group | Aboriginal and/or Torres Strait Islander peoples; Culturally and Linguistically Diverse; Rural or regional areas; LGBTQIA+ |
| Q20. Type of mental health problem for prevention | e.g. Psychological or psychosocial interventions for anxiety problems; Lifestyle interventions for ADHD |
| Q21. Type of intervention for prevention | Psychological or psychosocial; Pharmacological (i.e. medication); Complementary medicines; Lifestyle interventions; Devices |
| Q22. Other aspects of prevention trial design | Understanding the intervention components that have a positive impact (active ingredients, key mechanisms or mediators); Understanding what works for whom; Optimising existing prevention interventions (e.g. optimal dose/length, implementation); Side-effects/negative effects of prevention interventions; Trials focused on health economics of treatment – e.g. cost-effectiveness and cost-utility; Transdiagnostic approach to symptoms and co-occurring mental health disorders; Long term follow up for prevention trials (i.e. measuring if there is long-term improvement in mental health outcomes in children); Implementation of existing prevention interventions |
| **Section 4: Treatment trials** | |
| Q23. Setting of treatment trials | School/early childhood education; Community (e.g. sporting clubs, music or arts programs, play groups, churches); Health service; University (includes university-based clinics); Home – individual or family-based intervention; Online/digital |
| Q24. Type of mental health problem for treatment | Autism spectrum disorder; Communication disorders (speech or language disorders); Disruptive, impulse-control and conduct disorders; Dissociative disorders; Elimination disorders; Excessive crying disorders; Feeding and eating disorders; Intellectual Disabilities; Mood disorders (Bipolar, Depression); Motor disorders (e.g. developmental coordination disorder or Tourette's); Obsessive-compulsive disorders (OCD); Schizophrenia Spectrum & Other Psychotic Disorders; Self-harm; Sleep-wake or sleep disorders; Somatic symptom related disorders; Specific learning disorder; Substance-related and addictive disorders (including vaping); Trauma-and stressor-related disorders (e.g. PTSD) |
| Q25.Type of intervention for treatment | Psychological or psychosocial; Pharmacological (i.e. medication); Complementary medicines; Lifestyle interventions; Devices |
| Q26. Treatment intervention type for specific mental health problems | Psychological or psychosocial; Pharmacological (i.e. medication); Complementary medicines; Lifestyle interventions; Devices |
| Q27. Other aspects of treatment trial design | Understanding the intervention components that have a positive impact (active ingredients, key mechanisms or mediators); Understanding what works for whom;  Interventions for non-responders; Optimising existing treatment interventions (e.g. optimal dose/length, implementation); Side-effects/negative effects of prevention interventions; Trials focused on health economics of treatment – e.g. cost-effectiveness and cost-utility; Transdiagnostic approach to symptoms and co-occurring mental health disorders; Long term follow up for treatment trials (i.e. measuring if there is improvement mental health outcomes in children in the long term); Implementation of existing treatment interventions |
| **Section 5: Assessment trials** | |
| Q28. Screening and diagnostic tools | Improved screening tools; Improved diagnostic tools for specific disorders; Other assessment tools relevant to mental health promotion e.g. mental health literacy/stigma |
| Q29. Evaluating the impact of assessing child mental health indicators at routine developmental checks | Nil sub items for rating |
| Q30. What assessment tools are a priority for which mental health problems | e.g. Improved screening tools for anxiety;  Improved diagnostic tools for specific disorders such as eating disorders; Other assessment tools relevant to promotion (Mental Health Literacy/stigma) for ADHD. |

**Supplementary Table 2. Summary of 75 research topics and items endorsed as a priority at the end of Round 3. Items were endorsed if 80% of both panels rate them as either a “High” or “Very High” priority.**

| Topic and sub-items |
| --- |
| Section 1: High-level categories   - Age - Pre-school (3 - 4-9 years) - Age - Early primary school (5 - 8.9 years) - Age - Later primary school (9 - 12 years) - Child gender - Gender diverse (trans or non-binary) children - Priority populations - Aboriginal and/or Torres Strait Islander peoples - Priority populations - Rural or regional areas - Priority populations - Culturally and Linguistically Diverse - Priority populations - Mental health problems in children with neurodevelopmental disorders - Spectrum of intervention - Prevention - Spectrum of intervention – Treatment - Intervention participants – Children - Intervention participants - Parents and caregivers - Mental health problem - Anxiety disorders - Mental health problem - Co-occurring mental health problems - Other aspects of trial design - Real-world effectiveness trials: Evaluation of interventions in settings that reflect real-world practice - Other aspects of trial design - Consistent outcome measures for a range of domains, e.g. psychological distress, sleep, parent-child relationship, quality of life |
| Section 2: Promotion   - Reducing stigma - Parents/caregivers - Reducing stigma – Educators - Mental Health Literacy - Parents/caregivers - Mental Health Literacy – Educators - Improving the wellbeing of children experiencing mental health problems - Promotion trials by priority group - Aboriginal and Torres Strait Islander peoples - Promotion trials by priority group - Mental health problems in children with neurodevelopmental disorders - Promotion Setting - School/early childhood education - Long term follow up for promotion trials (i.e. measuring if there is long-term improvement in mental health outcomes in children) - Improving the wellbeing of parents/caregivers of children experiencing mental health problems |
| Section 3: Prevention   - Indicated prevention - Age of prevention; - Prevention in preschool age (3-4.9 years) - Age of prevention; - Prevention in early primary school age (5-8.9 years) - Age of prevention; - Prevention in late primary school age (9-12 years) - Age of prevention; - Prevention at transition points (i.e. from pre-school to primary school, or primary to secondary school) - Prevention setting - School/early childhood education - Prevention setting - Home – individual or family-based intervention - Prevention participants – Children - Prevention in Parents and caregivers - Prevention participants in Educators - Selective prevention of the following adverse childhood experience (ACE) - Children of parents with a mental illness - Selective prevention of the following ACE - Children who experience child abuse - Selective prevention of the following ACE - Children in out-of-home care / state care or subject to notifications of abuse - Selective prevention of the following ACE - Children who have been in contact with (or at risk of) the justice system - Selective prevention of the following ACE - Children with disabilities - Selective prevention of the following ACE - Children experiencing multiple adversities - Prevention by priority group - Aboriginal and Torres Strait Islander peoples - Prevention by priority group - Culturally and Linguistically Diverse - Prevention by priority group- Rural/Regional - Prevention trials - Anxiety disorders - Prevention trials - Trauma-and stressor-related disorders (e.g. PTSD) - Prevention - Self-harm and injury (non-suicidal behaviour) - Type of intervention for prevention - Psychological or psychosocial - Type of intervention for prevention - Interventions targeting the social determinants of health - Other aspects of prevention trial design - Understanding the intervention components that have a positive impact (active ingredients, key mechanisms or mediators) - Other aspects of trial design - Understanding what works for whom - Other aspects of trial design - Long term follow up for prevention trials (i.e. measuring if there is long-term improvement in mental health outcomes in children) |
| Section 4: Treatment   - Treatment setting - School/early childhood education - Treatment setting - Home – individual or family-based intervention - Treatment - Anxiety disorders - Treatment - Trauma-and stressor-related disorders (e.g. PTSD) - Treatment - Treatment for co-occurring mental health problems - Treatment - Self-harm and injury (non-suicidal behaviour) - Treatment - Suicide including thoughts and attempts - Treatment intervention type - Psychological or psychosocial - Psychological or psychosocial treatment interventions for anxiety - Psychological or psychosocial treatment interventions for mood disorders - Psychological or psychosocial treatment interventions for self-harm - Psychological or psychosocial treatment interventions for trauma-and stressor-related disorders (e.g. PTSD) - Other aspects of treatment trial design - Understanding the intervention components that have a positive impact (active ingredients, key mechanisms or mediators) - Other aspects of treatment trial design - Understanding what works for whom - Other aspects of treatment trial design - Long term follow up for treatment trials (i.e. measuring if there is improvement mental health outcomes in children in the long term) - Other aspects of treatment trial design- Transdiagnostic approach to symptoms and co-occurring mental health disorders |
| Section 5: Assessment   - Screening and diagnostic tools - Improved screening tools (0-12 years) - Screening and diagnostic tools - Improved screening tools for younger children (0 - 8.9 years) - Screening and diagnostic tools- Improved diagnostic tools for mental health problems in children with neurodevelopmental disorders - Evaluating the impact of assessing child mental health indicators at routine developmental checks - Trials evaluating linking screening with appropriate care |

**Supplementary Table 3**. Results of rounds 1-3 rating by panel

<See attachment>

**Supplementary Table 4. Summary of the topics and items endorsed by 85% or more of both panels at the end of Round 3.**

| **Topic Reference Number** | **Topic - Item** | **Professional Panel (High + Very high priority) %** | **Lived Experience Panel (High + Very high priority) %** |
| --- | --- | --- | --- |
| **Section 1** | | | |
| **Q1** | Child Age - Later primary school (9 - 12 years) | 89.7 | 88.9 |
| **Q3** | Priority populations - Aboriginal and/or Torres Strait Islander peoples | 87.2 | 85.2 |
| **Q3** | Priority populations - (NEW) Mental health problems in children with neurodevelopmental disorders | 88.6 | 96.2 |
| **Q4** | Spectrum of intervention - Prevention | 85.7 | 88.5 |
| **Q5** | Intervention participants - Parents and caregivers | 92.3 | 100.0 |
| **Q7** | Real-world effectiveness trials - Evaluation of interventions in settings that reflect real-world practice | 92.3 | 92.6 |
| **Section 2** | | | |
| **Q9** | Mental health literacy - Parents/caregivers | 94.9 | 96.3 |
| **Q9** | Mental health literacy - Educators | 94.9 | 92.6 |
| **Q10** | Improving the wellbeing of children experiencing mental health problems | 87.2 | 92.6 |
| **Q11** | Promotion trials - Aboriginal and Torres Strait Islander peoples | 87.2 | 85.2 |
| **Q13** | Long term follow up for promotion trials (i.e. measuring if there is long-term improvement in mental health outcomes in children) | 89.7 | 88.9 |
| **Section 3** | | | |
| **Q14** | Indicated prevention | 92.3 | 85.2 |
| **Q15** | Age of prevention; - Prevention in early primary school age (5-8.9 years) | 94.9 | 88.9 |
| **Q15** | Age of prevention; - Prevention in late primary school age (9-12 years) | 87.2 | 96.3 |
| **Q15** | Age of prevention; - Prevention at transition points (i.e. from pre-school to primary school, or primary to secondary school) | 89.7 | 96.3 |
| **Q16** | Prevention setting - School/early childhood education | 94.9 | 85.2 |
| **Q16** | Prevention setting - Home – individual or family-based intervention | 88.6 | 96.2 |
| **Q17** | Prevention in Parents and caregivers | 94.9 | 100.0 |
| **Q18** | Selective prevention (ACE) - Children who experience child abuse | 92.3 | 100.0 |
| **Q18** | Selective prevention (ACE) - Children in out-of-home care / state care or subject to notifications of abuse | 94.9 | 92.6 |
| **Q18** | Selective prevention (ACE) - Children who have been in contact with (or at risk of) the justice system | 89.7 | 88.9 |
| **Q18** | Selective prevention (ACE) - Children with disabilities | 85.7 | 88.5 |
| **Q18** | Selective prevention (ACE) - (NEW) Children experiencing multiple adversities | 91.4 | 92.3 |
| **Q19** | Prevention by priority group - Aboriginal and Torres Strait Islander peoples | 87.2 | 88.9 |
| **Q20** | Prevention trials - Anxiety disorders | 92.3 | 96.3 |
| **Q20** | Prevention trials - Trauma-and stressor-related disorders (e.g. PTSD) | 87.2 | 92.6 |
| **Q20** | Prevention - (NEW) *Self-harm and injury (non-suicidal behaviour) | 85.7 | 96.2 |
| **Q21** | Type of intervention for prevention - Psychological or psychosocial | 89.7 | 96.3 |
| **Q22** | Other aspects of prevention trial design - Understanding the intervention components that have a positive impact (active ingredients, key mechanisms or mediators) | 94.9 | 85.2 |
| **Q22** | Other aspects of trial design - Understanding what works for whom | 94.9 | 100.0 |
| **Q22** | Other aspects of trial design - Long term follow up for prevention trials (i.e. measuring if there is long-term improvement in mental health outcomes in children) | 87.2 | 100.0 |
| **Section 4** | | | |
| **Q24** | Treatment - Anxiety disorders | 87.2 | 92.6 |
| **Q24** | Treatment - (NEW) *Suicide including thoughts and attempts | 87.1 | 92.0 |
| **Q24** | Treatment - (NEW) Treatment for co-occurring mental health problems | 85.7 | 88.5 |
| **Q25** | Treatment intervention type - Psychological or psychosocial | 94.9 | 96.3 |
| **Q27** | Other aspects of treatment trial design - Understanding the intervention components that have a positive impact (active ingredients, key mechanisms or mediators) | 94.9 | 85.2 |
| **Q27** | Other aspects of treatment trial design - Understanding what works for whom | 94.9 | 92.6 |
| **Section 5** | | | |
| **Q29** | Evaluating the impact of assessing child mental health indicators at routine developmental checks | 87.2 | 88.9 |

**Supplementary Table 5.1 Results of Round 4 by Panel -** **Summary of Round 4 Results for Topic 1: Promotion Trials.**

Priorities above the weighted mean (14.28) are highlighted in green.

| Topic Reference Number | Topic - Item | Mean (both groups) | Mean  (LE Panel) | Mean (Prof Panel) |
| --- | --- | --- | --- | --- |
| Q1_3 | Improving the wellbeing of children experiencing mental health problems | **22.28** | 22.88 | 21.67 |
| Q1_1 | Improving mental health literacy in parents/caregivers | **17.44** | 15.58 | 19.31 |
| Q1_5 | Promotion by priority group - Mental health problems in children with neurodevelopmental disorders | **13.81** | 17.50 | 10.12 |
| Q1_7 | 7. Other aspects of promotion trial design - Real-world effectiveness promotion trials: Evaluation of interventions in settings that reflect real-world practice | **13.32** | 12.88 | 13.76 |
| Q1_4 | 4. Promotion by priority group - Aboriginal and Torres Strait Islander peoples | **10.63** | 7.88 | 13.38 |
| Q1_2 | 2. Improving mental health literacy in educators | **9.71** | 14.42 | 5.00 |
| Q1_6 | 6. Other aspects of promotion trial design - Long term follow up for promotion trials (i.e. measuring if there is long-term improvement in mental health outcomes in children) | **9.42** | 8.85 | 10.00 |

**Supplementary Table 4.2 Results of Round 4 by Panel** - **Summary of Round 4 Results for Question 2: Prevention and Assessment Trials.**

Priorities above the weighted mean (5.26) are highlighted in green.

| Topic Reference Number | Topic - Item | **Mean (both groups)** | **Mean (LE Panel)** | **Mean (Prof Panel)** |
| --- | --- | --- | --- | --- |
| Q2_8 | Selective prevention in at risk population groups for one of the following adverse childhood events: *Children who experience child abuse; children in out-of-home care / state care or subject to notifications of abuse; children in contact with (or at risk of) the justice system; children with disabilities; or children experiencing multiple adversities.* | **12.43** | 13.79 | 11.08 |
| Q2_1 | Age - Prevention in early primary school age (5-8.9 years) | **9.66** | 7.17 | 12.15 |
| Q2_18 | Evaluating the impact of assessing child mental health indicators at routine developmental checks (early intervention & prevention) | **7.23** | 8.83 | 5.63 |
| Q2_3 | Age - Prevention at transition points (i.e. from pre-school to primary school, or primary to secondary school) | **6.80** | 5.96 | 7.65 |
| Q2_2 | Age - Prevention in late primary school age (9-12 years) | **6.39** | 6.25 | 6.53 |
| Q2_5 | Prevention setting - Home: individual or family-based intervention | **5.64** | 4.75 | 6.53 |
| Q2_19 | Other aspects of prevention trial design - Real-world effectiveness prevention trials: Evaluation of interventions in settings that reflect real-world practice | **5.33** | 6.79 | 3.88 |
| Q2_10 | Prevention by priority group - Mental health problems in children with neurodevelopmental disorders | **5.18** | 7.08 | 3.28 |
| Q2_4 | Prevention setting - School/early childhood education | **5.13** | 5.46 | 4.80 |
| Q2_6 | Prevention trials in parents and caregivers | **4.99** | 2.13 | 7.85 |
| Q2_12 | Prevention trials for Trauma-and stressor-related disorders (e.g. PTSD) | **4.51** | 7.13 | 1.90 |
| Q2_17 | Other aspects of trial design - Long term follow up for prevention trials (i.e. measuring if there is long-term improvement in mental health outcomes in children) | **4.41** | 2.75 | 6.08 |
| Q2_9 | Prevention by priority group - Aboriginal and Torres Strait Islander peoples | **4.14** | 3.08 | 5.20 |
| Q2_16 | Other aspects of trial design - Understanding what works for whom | **3.81** | 3.79 | 3.83 |
| Q2_14 | Psychological or psychosocial prevention interventions | **3.59** | 4.50 | 2.68 |
| Q2_15 | Other aspects of prevention trial design - Understanding the intervention components that have a positive impact (active ingredients, key mechanisms or mediators) | **3.28** | 2.96 | 3.60 |
| Q2_13 | Prevention trials for Self-harm and injury (non-suicidal behaviour) | **3.06** | 4.29 | 1.83 |
| Q2_11 | Prevention trials for Anxiety disorders | **2.58** | 1.96 | 3.20 |
| Q2_7 | Indicated prevention | **1.84** | 1.33 | 2.35 |

**Supplementary Table 5.2 Results of Round 4 by Panel** - **Summary of Round 4 Results for Question 3: Treatment Trials.**

Priorities above the weighted mean (14.28) are highlighted in green.

| Topic Reference Number | Topic - Item | **Mean (both groups)** | **Mean**  **(LE Panel)** | **Mean (Prof Panel)** |
| --- | --- | --- | --- | --- |
| Q3_4 | Other aspects of treatment trial design - Real-world effectiveness treatment trials: Evaluation of interventions in settings that reflect real-world practice | **18.10** | 16.33 | 19.88 |
| Q3_6 | Treatment of co-occurring mental health problems | **16.50** | 20.71 | 12.30 |
| Q3_1 | Other aspects of treatment trial design - Understanding the intervention components that have a positive impact (active ingredients, key mechanisms or mediators) | **15.30** | 13.21 | 17.40 |
| Q3_3 | Other aspects of treatment trial design - Understanding what works for whom | **14.80** | 11.46 | 18.15 |
| Q3_5 | Treatment of suicide including thoughts and attempts | **12.78** | 17.38 | 8.18 |
| Q3_7 | Treatment of anxiety disorders | **11.86** | 11.54 | 12.18 |
| Q3_2 | Treatment intervention type - Psychological or psychosocial treatment interventions | **10.65** | 9.38 | 11.93 |
